# Three-dimensional representation of triple spectral line imaging data as an option for noncontact skin diagnostics

**DOI:** 10.1117/1.JBO.27.9.095005

**Published:** 2022-09-16

**Authors:** Ilze Oshina, Janis Spigulis, Ilona Kuzmina, Laura Dambite, Anna Berzina

**Affiliations:** a University of Latvia, Biophotonics Laboratory, Institute of Atomic Physics and Spectroscopy, Riga, Latvia; b University of Latvia, Physics Department, Faculty of Physics, Mathematics and Optometry, Riga, Latvia; c The Clinic of Laser Plastics, Riga, Latvia

**Keywords:** optical skin diagnostics, spectral line imaging, skin chromophores, image processing, Beer–Lambert law

## Abstract

**Significance:**

Skin malformations in dermatology are mostly evaluated subjectively, based on a doctor’s experience and visual perception; an option for objective quantitative skin assessment is camera-based spectrally selective diagnostics. Multispectral imaging is a technique capable to provide information about concentrations of the absorbing chromophores and their distribution over the malformation in a noncontact way. Conversion of spectral images into distribution maps of chromophores can be performed by means of the modified Beer–Lambert law. However, such distribution maps represent only single specific cases, therefore, some extensive method for data comparison is needed.

**Aim:**

This study aims to develop a more informative approach for identification and characterization of skin malformations using three-dimensional (3D) representation of triple spectral line imaging data.

**Approach:**

The 3D-representation method is experimentally tested on eight different skin pathology types, including both benign and malignant pathologies; an imaging device ensuring uniform three laser line (448, 532, and 659 nm) illumination is used. Three spectral line images are extracted from a single snapshot RGB image data, with subsequent calculation of attenuation coefficients for each working wavelength at every image pixel and represented as 3D graphs. Skin chromophore content variations in malformations are represented in a similar way.

**Results:**

Clinical measurement results for 99 skin pathologies, including basal cell carcinomas, melanoma, dermal nevi, combined nevi, junctional nevi, blue nevi, seborrheic keratosis, and hemangiomas. They are presented as 3D spectral attenuation maps exhibiting specific individual features for each group of pathologies. Along with intensity attenuation maps, 3D maps for content variations of three main skin chromophores (melanin, oxyhemoglobin, and deoxyhemoglobin), calculated in frame of a model based on modified Beer–Lambert law, are also presented. Advantages and disadvantages of the proposed data representation method are discussed.

**Conclusions:**

The described 3D-representation method of triple spectral line imaging data shows promising potential for objective quantitative noncontact diagnosis of skin pathologies.

## 1 Introduction

To ensure reliable and accurate diagnostics of skin malformations, direct *in-vivo* measurements of skin chromophore content variations and distributions across the lesion area are of great clinical value, especially if the measurements are taken in a patient-friendly noninvasive way. One possible approach is multispectral imaging of skin diffuse reflectance where the specular reflection of skin is minimized by using two orthogonally oriented polarizers – one in front of the image sensor (camera) and the second in front of the light source.^1^ To extract the chromophore concentrations or their changes in particular skin image areas, the Beer–Lambert law (BLL) can be used.^2^^–^^5^ However, BLL applied for skin diffuse reflectance may also lead to some uncertainties and bottlenecks;^6^ therefore, attention should be paid to specific parameters used in calculation models for image processing. Applicability of a modified BLL for evaluation of skin chromophore content changes in malformations using pixel-by-pixel analysis of diffusely reflected light intensity at fixed wavelengths is tested in this study.

The potential of the snapshot triple spectral line imaging method for noncontact assessment of various skin malformations has been successfully demonstrated in Refs. 1, 2, and 7. This study continues our previous research with an extended number of clinical cases (99 in total); a novel approach for presenting spectral line image processing results using three-dimensional (3D) representation of the imaging data is introduced.

## 2 Methods

Skin can be modeled as a structure comprising a number of homogeneous layers. In simple models only two layers are considered: epidermis and dermis,^8^ while in more advanced models up to seven layers are taken into account: stratum corneum, living epidermis, papillary dermis, papillary plexus, reticular dermis, cutaneous plexus, and subcutis.^9^ Each skin layer has specific optical properties – absorption coefficient, anisotropy factor, refractive index, as well as own thickness and chromophore composition. Besides, skin scattering properties are to be taken into account.

### 2.1 Model

A modified BLL model for diffusely reflected light was tested experimentally in this work. Reflected intensity from the surrounding healthy skin was always taken as a reference in analysis of the malformation-reflected light intensity;^1^ therefore, all presented skin chromophore concentration values in pathologies are relative compared with the volunteers healthy skin. Exact values for mean path lengths of the skin-remitted photons at particular wavelengths are needed for processing the measured data. Here, we used experimentally determined values from our previous study of the photon time-of-flight in skin.^10^ In this study, picosecond laser pulses at eight narrow wavelength bands were launched into healthy forearm skin of volunteers via an optical fiber, and the diffusely reflected signals were detected at various distances by means of the time-correlated single-photon counting method. By comparing the shapes of input and output pulses [a(t) and b(t)], the temporal distribution function f(t) of photon arrivals following infinitely narrow δ-pulse input was found by deconvolution of the integral,

$$b(t)=\int_{0}^{t}a(t-\tau )f(\tau )d\tau .$$ (1)

The corresponding distribution of the backscattered photon path lengths in skin was found as

ϕ(s)=f(t)·c/n, (2)

where c is the speed of light in vacuum, n is the averaged refraction index of superficial skin tissues (∼1.4). Photon means path length in skin was calculated as the mean value of integrated path length distribution function.

It was assumed that only skin melanin, oxyhemoglobin, and deoxyhemoglobin are absorbing the incident light in this model. Scattering was excluded because diffusely reflected light intensities from the skin pathology and the healthy skin were compared, assuming that scattering properties in the healthy skin and the pathology region are very similar. Reduced scattering coefficient ${\mu_{s}}^{\prime }$ values can change up to two times^11^ which does not contribute much to the backscattered light intensity, if compared with chromophore absorption. Modified BLL model for diffusely reflected light:

$$c_{\mathrm{Mel}}\cdot \varepsilon_{\mathrm{Mel}}(\lambda )+c_{\mathrm{Ox}}\cdot \varepsilon_{\mathrm{Ox}}(\lambda )+c_{\mathrm{Deox}}\cdot \varepsilon_{\mathrm{Deox}}(\lambda )=\frac{\ln\frac{I_{0}(\lambda )}{I(\lambda )}}{2.303l(\lambda )},$$ (3)

where Mel is the melanin; Ox is the oxyhemoglobin; Deox is the deoxyhemoglobin; ε(λ) is the extinction coefficient (${\mathrm{cm}}^{-1}/M$);^12^^,^^13^ c is changed chromophore concentration in the pathology region (M); I(λ) and $I_{0}(\lambda )$ are the intensities of diffusely reflected (remitted) light from the pathology and from the surrounding skin (relative units), respectively; and l(λ) (cm) is remitted photon mean path length in the skin at particular wavelength λ. We use three wavelengths for skin illumination, therefore, we can write Eq. (1) three times obtaining a system of three Eq. (1). There are three unknown values $c_{\mathrm{Mel}}$, $c_{\mathrm{Ox}}$, and $c_{\mathrm{Deox}}$ in this system. Accordingly, we can calculate variations of all chromophore concentrations by solving this system of equations.

### 2.2 Measurement Setup

**Fig. 1:**
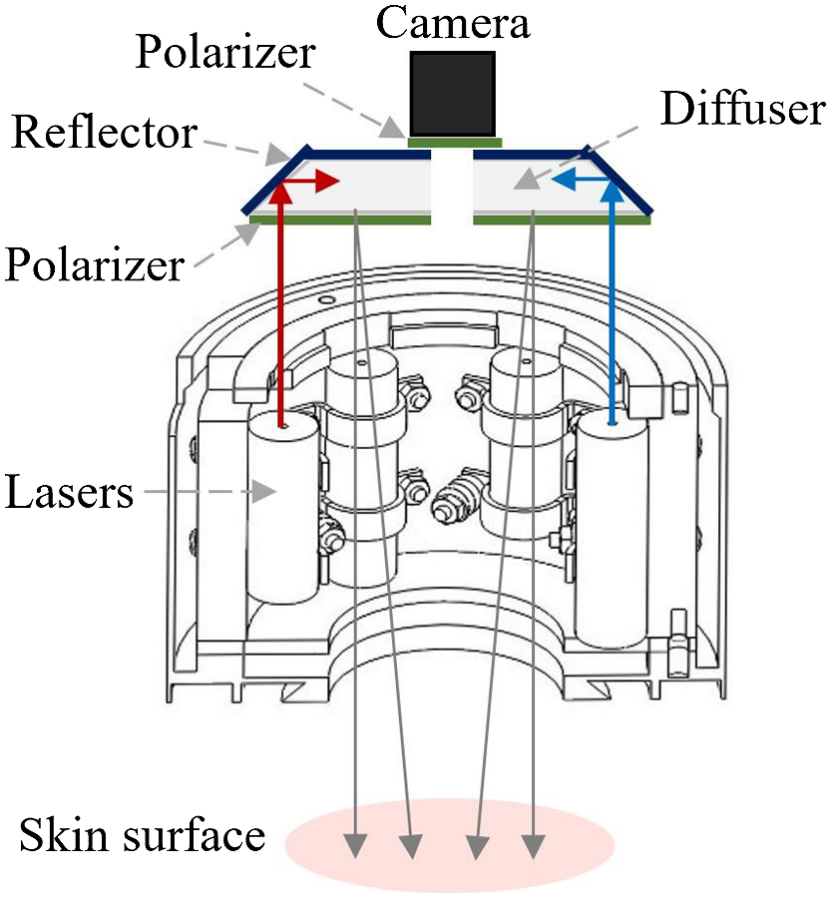
Illumination and image capturing scheme of the experimental device.^14^

Our previously created device^1^ was used for clinical measurements; the setup scheme is presented in Fig. 1. Skin was uniformly illuminated by three narrow laser-emitted spectral lines using a specially designed disc-shaped light diffuser. Three couples of laser modules with equal emission wavelengths were placed at opposite sides of the light-shielding cylindrical wall of the device, so six radial laser beams were scattered by the diffuser. Three laser lines (448, 532, and 659 nm) via the diffusive reflector simultaneously illuminated the skin region of interest comprising the skin malformation – e.g., nevus, hemangioma, or seborrheic keratosis. RGB camera of a Nexus 5 smartphone was used for spectral image data collection using AZ Camera software. Two orthogonally oriented polarizers were exploited to minimize detection of skin specular reflection – one in front of illumination source and another in front of the camera. The single snapshot approach with exposure time in the millisecond range excluded the motion artifacts in three spectral line images.

### 2.3 Image Processing

**Fig. 2:**
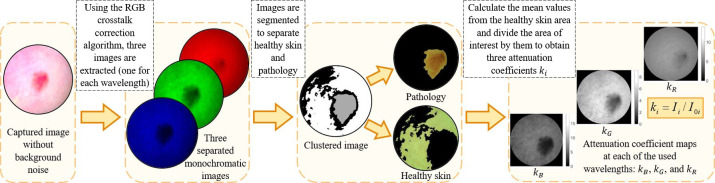
Image processing scheme.

Image processing scheme is illustrated in Fig. 2. When transforming a single RGB image data set (taken under three spectral line illumination) into three spectral line images – one image for each laser line, the RGB crosstalk correction is necessary. The correction algorithm Ref. 15 exploits three spectral sensitivity curves (R, G, and B) of the image sensor. Crosstalk means that each wavelength can be detected at two or three detection bands of the sensor, but with different probabilities – depending on the detection band sensitivity for this wavelength. The crosstalk correction algorithm extracts the contribution of each working wavelength in the output signal of each detection band (R, G, and B). In our case, skin was uniformly illuminated by three laser lines (448, 532, and 659 nm) and we used the provided manufacturer spectral sensitivity values at the R, G, and B detection bands of the sensor for these wavelengths for crosstalk correction. Sets of R, G, and B channel values were collected from each pixel, then the crosstalk correction was performed with further calculations of the corresponding three spectral line images. These images were segmented to separate the pathology from healthy skin area using k-means clustering function in MATLAB.^16^ The green 532-nm image was used for segmentation. After that, the mean reflected intensity values from the surrounding healthy skin area at each of the used wavelengths ($I_{0}(\lambda )$) were calculated; they represented reference values when there were no tissue absorption changes due to skin pathologies. The reflected spectral line intensities from image pixels related to the area of pathology were divided by the reference values to obtain three attenuation coefficients $k(\lambda )=I(\lambda )/I_{0}(\lambda )$ (Fig. 2), where $k_{B}$ is related to 448 nm, $k_{G}$ to 532 nm, and $k_{R}$ to 659 nm wavelength.

### 2.4 Clinical Measurement Conditions

Clinical *in-vivo* measurements were taken under permission of the local ethics committee having written consent of all 77 volunteers with skin photo-types I or II (Fitzpatrick classification), aged between 20 and 68, in cooperation with certified dermatologist Anna Berzina (The Clinic of Laser Plastics, Riga). In total, 99 skin pathologies were examined in this study: three basal cell carcinomas, 27 dermal nevi, 12 hemangiomas, 16 combined nevi, one melanoma, 17 junctional nevi, 22 seborrheic keratoses, and one blue nevus. Data for dermal, combined, and junctional nevi, as well as those for hemangiomas and seborrheic keratoses, may be considered as sufficient for primary conclusions while the few cases of skin cancers (basal cell carcinoma and melanoma), and blue nevus can serve only for general illustration.

## 3 Results

### 3.1 3D Graphs for Spectral Attenuation Coefficients

**Fig. 3:**
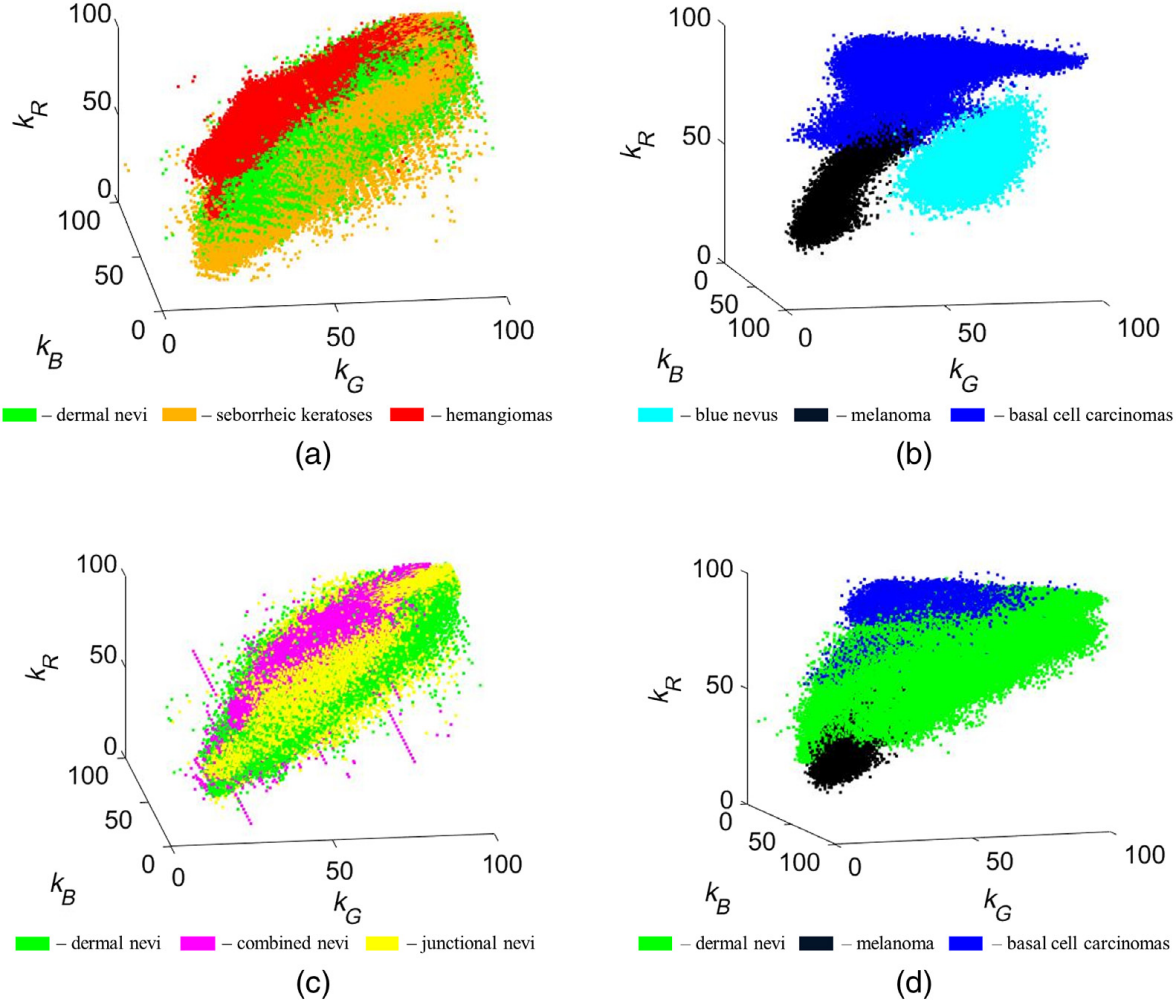
Attenuation coefficient 3D graphs in % for different groups of skin pathologies: (a) dermal nevi, seborrheic keratoses, and hemangiomas; (b) blue nevus, melanoma, and basal cell carcinomas; (c) dermal, combined, and junctional nevi; (d) dermal nevi, melanoma, and basal cell carcinomas.

The measured attenuation coefficients $k_{i}$ (reflected intensity ratios: pathology versus healthy skin) for the above-mentioned eight groups of pathologies are presented as 3D graphs in Fig. 3; all $k_{i}$ values are calculated in percentages. Each point in the graph represents one clinical data pixel value from a segmented pathology. Points are arranged more densely where pixels have similar values, and more scattered where only few pixels have these values. For some pathology types, like dermal nevi, we had more samples (27); therefore, 3D graph cloud consists of 400,000 nonoverlapping points. But for other pathologies, like blue nevi and melanoma, we had only one sample each, therefore, 3D graph cloud consists just of 6000 nonoverlapping points.

Figure 3(a) compares the k-value clouds for three different benign pathologies – dermal nevi, seborrheic keratoses, and hemangiomas. Parts of them are nonoverlapping, e.g., the specific volume related to hemangiomas and that related to nevi can be easily distinguished. Even better separation between malformations can be observed in Fig. 3(b) where spectral attenuations of two malignant pathologies – melanoma and basal cell carcinomas – are compared with those of a benign pathology – blue nevus. All three pathologies here can easily be distinguished; the $k_{R}$ values for blue nevus and melanoma are lower than those for basal cell carcinomas while melanoma exhibits lower $k_{G}$ values than the blue nevus. Spectral attenuations of three different nevi types – dermal, combined, and junctional – are compared in Fig. 3(c); they form a compact cloud but still, each type mainly covers a specific volume in the 3D graph. Data for malignant pathologies (basal cell carcinomas and melanoma) and typical benign pathology (dermal nevi) are compared in Fig. 3(d). Again, some values of all three malformations are slightly overlapping but the $k_{R}$ values for nevi are clearly higher than those for melanoma and lower than those for basal cell carcinomas.

To summarize, the graphs in Fig. 3 exhibit specific volume-shape features for each of the examined eight groups of skin pathologies. This kind of image data representation may find further application in quantitative diagnostics of skin malformations, e.g., for characterizing and identifying of specific skin pathologies by the spatial location of the $k_{B}-k_{G}-k_{R}$ data points determined from the spectral line images.

### 3.2 Attenuation Coefficient Cubes and Limiting Values for Skin Chromophore Content Changes

**Fig. 4:**
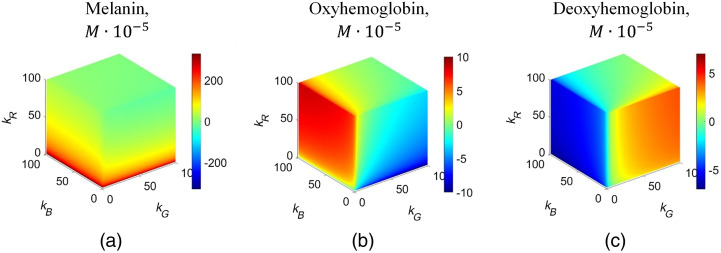
Full 3D cubes representing possible concentration variations of three main skin chromophores, calculated in frame of the used modified BLL model: (a) melanin, (b) oxyhemoglobin, and (c) deoxyhemoglobin. Color bar, the chromophore concentration increase/decrease in the malformation, expressed in moles $\cdot 10^{-5}$.

All possible values of chromophore concentration variations for melanin, oxyhemoglobin, and deoxyhemoglobin in moles (M) were calculated for all possible attenuation coefficient values from 1% to 100% (full cubes) in frame of the above-regarded modified BLL model (Sec. 2.1.) using the 3D data representation (Fig. 4). Those values can be both positive (increased chromophore concentration) and negative (decreased concentration) since they represent chromophore concentration changes in pathology compared with surrounding healthy skin (shown in the color bars of Fig. 4). Melanin concentration increase in pathology is inversely correlated to the $k_{R}$ values [Fig. 4(a)], as extinction coefficient of melanin at 659 nm is considerably lower than that at the two other used wavelengths. The color changes (representing concentration increase/decrease changes) in the oxyhemoglobin and deoxyhemoglobin cubes are almost reverse: where oxyhemoglobin values are higher, deoxyhemoglobin values are lower, and vice versa [Figs. 4(b) and 4(c)].

The model-limited chromophore content variation ranges were calculated, as well (Table 1). Variation range for melanin was the largest, from −23.67 to 338.93 $M\cdot 10^{-5}$. Oxy- and deoxy-hemoglobin concentration variations were model-limited in much narrower ranges.

**Table 1:** Minimal, maximal values, and value ranges for melanin, oxyhemoglobin, and deoxyhemoglobin concentration changes in skin malformations according to the used modified BLL model, $k_{i}\in [1\%,100\%]$.

Melanin, $M\cdot 10^{-5}$			Oxyhemoglobin, $M\cdot 10^{-5}$			Deoxyhemoglobin, $M\cdot 10^{-5}$		
Min	Max	Range	Min	Max	Range	Min	Max	Range
−23.67	338.93	362.60	−10.36	8.56	18.92	−7.15	4.20	11.35

### 3.3 3D Representation of the Clinical Data

**Fig. 5:**
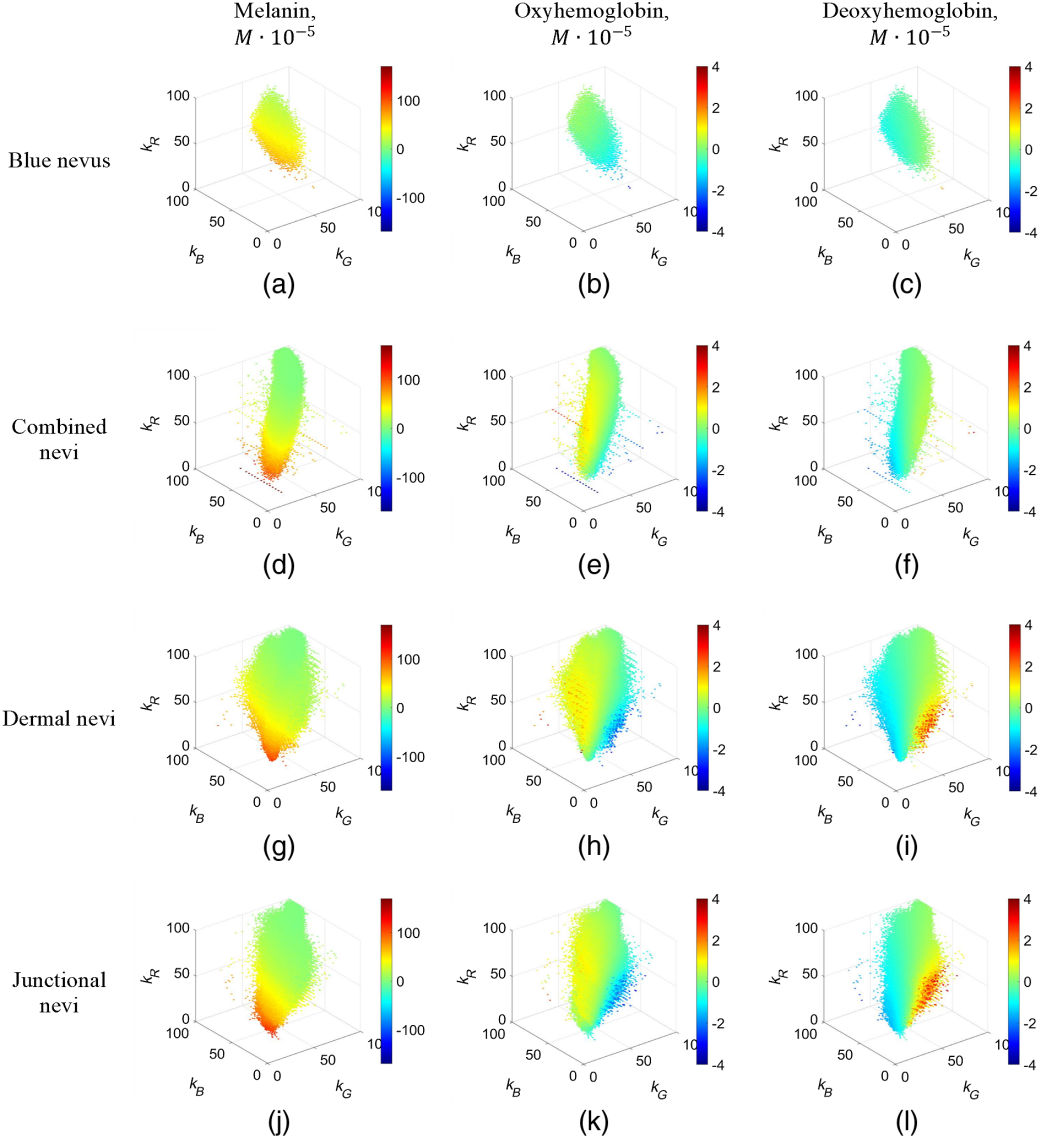
3D representation of chromophore content changes in nevi: (a)–(c) blue nevus, (d)–(f) combined nevi, (g)–(i) dermal nevi, and (j)–(l) junctional nevi. Color bar, the chromophore concentration increase/decrease in the pathologies, moles $\cdot 10^{-5}$.

**Fig. 6:**
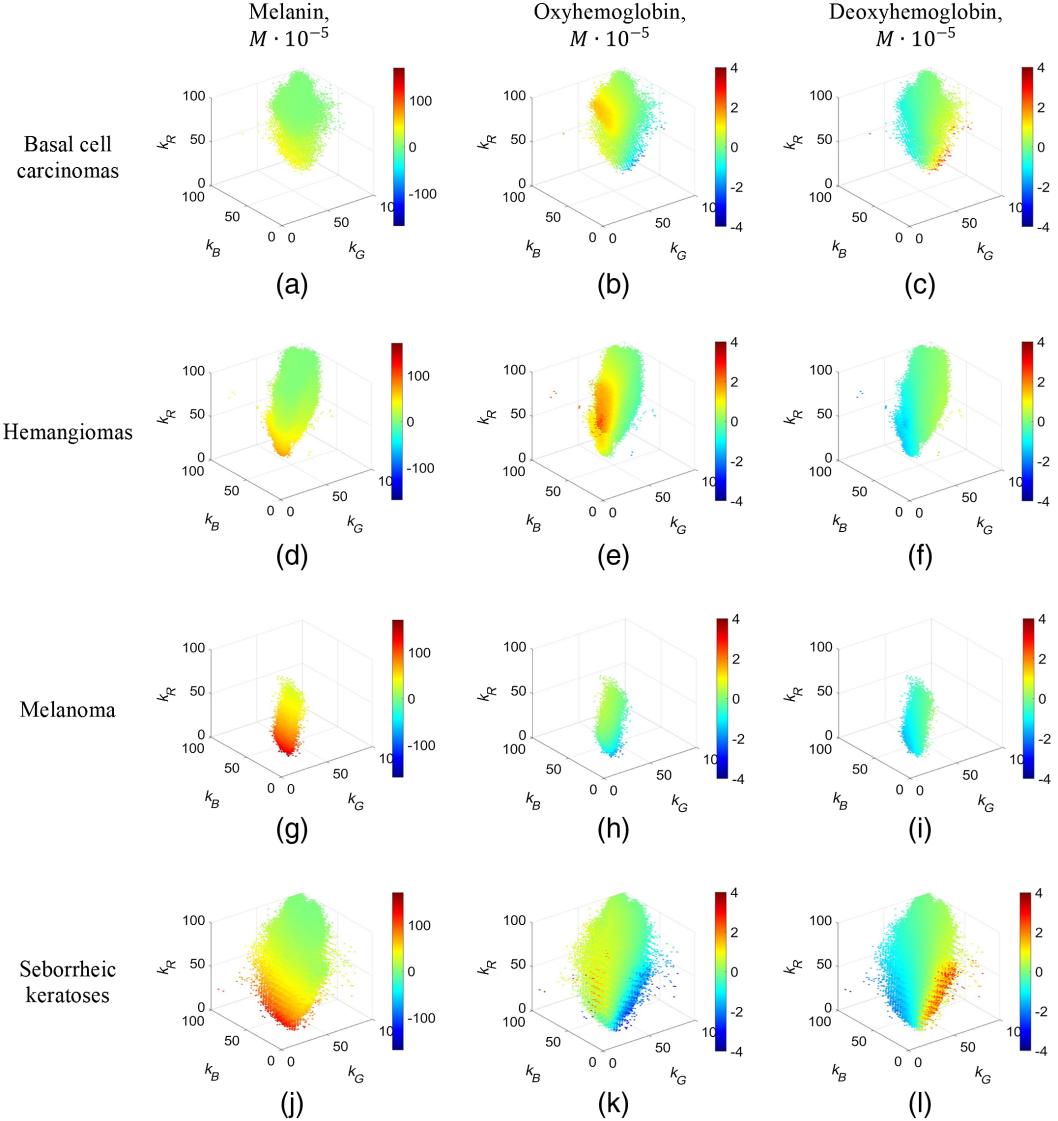
3D representation of chromophore content changes in four examined skin pathologies: (a)–(c) basal cell carcinomas, (d)–(f) hemangiomas, (g)–(i) melanoma, and (j)–(l) seborrheic keratoses. Color bar, the chromophore concentration increase/decrease in the pathologies, moles $\cdot 10^{-5}$.

Four groups of nevi were analyzed using the above-described 3D representation: blue, combined, dermal, and junctional (Fig. 5). As well as four other groups of pathologies: basal cell carcinomas, hemangiomas, melanoma, and seborrheic keratoses (Fig. 6). Only points representing the pixel values in clinical data images were left in the 3D graphs of spectral attenuation coefficients. Green color in all graphs represents chromophore zero changes. If relative chromophore content is higher in the pathology than in the adjacent healthy skin, the points in the graph are colored yellow or red; if the chromophore content has decreased, the points are colored blue. The melanin, oxyhemoglobin, and deoxyhemoglobin relative concentration scales are leveled equally for all included pathologies.

Different specific spatial cloud shapes in the presented 3D graphs are related to the examined skin pathologies, along with different k-value distributions. Nevi and melanoma exhibit the highest melanin concentration increase values [Figs. 5(a), 5(d), 5(g), 5(j), and 6(g)] while the oxyhemoglobin concentration increase values are highest for hemangiomas [Fig. 6(e)], as could be expected from the lesion anatomy considerations.

## 4 Discussion

A novel approach for skin pathology analysis using sets of triple spectral line images has been proposed and tested. The clinical image data were compiled in 3D intensity-attenuation coefficient graphs at three fixed wavelengths 448, 532, and 659 nm (Figs. 3, 5, and 6). Although some of the obtained 3D clouds for particular skin malformations spatially overlapped (Fig. 3), individual features of specific pathology groups like cloud shape/volume and chromophore content distribution could be clearly distinguished. The spectral imaging data were processed in frame of a modified BLL model for diffusely reflected light where only relative concentration changes of three main skin chromophores (melanin, oxyhemoglobin, and deoxyhemoglobin) with respect to the surrounding healthy skin were calculated. In spite of evident limitations of the used model, the proposed 3D representation approach demonstrated not only selectivity concerning the type of pathology but also some advantages if compared to the previously used approach – planar mapping of the chromophore distributions over the skin malformation.^1^^,^^2^^,^^5^^,^^17^^–^^20^ The planar chromophore maps are well-suited for assessment of a specific single pathology sample, but this representation lacks sense of general trends of chromophore distributions in the pathology groups. Qualitatively, the main criteria for the first stage study are fulfilled: all hemangiomas have exhibited higher oxyhemoglobin concentration increase than other examined pathologies and all pigmented malformations (nevi, basaliomas, and melanoma) have exhibited higher melanin concentration increase.

The 3D representation of spectral imaging data potentially enables objective comparison of different pathology groups and may facilitate a general understanding of the chromophore content variations and their distributions in skin malformations. Authors believe that further development of this approach, e.g., by applying AI algorithms for the 3D cloud recognition, could be helpful for improved quantitative diagnostics of particular pathologies, including skin cancers, as well as for reliable follow-up of the skin healing process after therapies.

As a disadvantage, the need for specific equipment for spectral line imaging of skin can be mentioned. However, currently, there are all technical preconditions for creation of suitable low-cost diagnostic devices of this kind as compact and stable low-power RGB lasers are entering the market^21^ and uniform triple spectral line illumination of skin can be easily performed, e.g., by exploiting side-emitting optical fibers.^22^ Tools for elimination of the laser speckle artifacts in spectral line images are developed, as well.^23^

Concerning further developments, some more advanced modifications of BLL-based image processing models taking into account absorption/scattering in several skin layers have to be tested in conjunction with the 3D representation of spectral attenuation coefficients. Besides, the mean path lengths of skin-remitted photons from various skin malformations have to be determined and used in model calculations, instead of the data for healthy skin exploited in this paper. Larger groups of volunteers should be clinically inspected in future, including those with darker skin in order to examine skin type impact on calculated chromophore concentration values.

## 5 Summary

Results of a clinical study involving eight types of skin malformations (99 in total) by means of the triple spectral line snapshot imaging method were represented as 3D plots of spectral attenuations, so demonstrating specific shape/volume features of each pathology group. Concentration variations of three main skin chromophores in the examined malformations were calculated in frame of a modified Beer–Lambert model and 3D-represented, as well. Further development of this technique may contribute to improved noncontact objective diagnostics of skin pathologies, including skin cancers.

## Code, Data, and Materials Availability

The code and data underlying the results presented in this paper are not publicly available at this time but may be obtained from the authors upon reasonable request. No materials were used for the analysis.
